# Experiences of suicide survivors of sharing their stories about suicidality and overcoming a crisis in media and public talks: a qualitative study

**DOI:** 10.1186/s12889-024-17661-4

**Published:** 2024-01-10

**Authors:** Stefanie Kirchner, Thomas Niederkrotenthaler

**Affiliations:** 1 Unit Suicide Research & Mental Health Promotion, Department of Social and Preventive Medicine, Center for Public Health, Medical University of Vienna, Kinderspitalgasse 15/1, Vienna, 1090 Austria; 2 Wiener Werkstaette for Suicide Research, Vienna, Austria

**Keywords:** Lived experience, Suicide, Media, Storytelling, Papageno effect, Suicide prevention

## Abstract

**Background:**

Media stories of hope and recovery from suicidal ideation have been found to have a positive impact on the audience, but little is known about how individuals who share their own experiences perceive the effects of their storytelling. This study aimed to assess qualitatively, through focus groups, how individuals who shared their personal story of hope and recovery in the media and public talks experienced the process, and which aspects they perceived as important in sharing their coping story.

**Methods:**

Three focus groups were conducted with a total of *n* = 12 individuals. These included *n* = 5 participants with experience of suicidal ideation or a suicide attempt, *n* = 4 individuals who had been bereaved through suicide, and *n* = 3 participants who experienced both. Participants were recruited from the American organisation “Suicide Survivors United”. Thematic analysis was used to assess the participants’ perception and experiences of sharing their story.

**Results:**

Participants shared that the intention to help others was the main motivation to share their story of hope and recovery. Participants noted many positive effects of their storytelling on themselves and also received positive feedback from the audience, such as improved help-seeking attitudes. The participants offered recommendations for those who want to share their story of hope and recovery, including careful personal preparation and media training before going public. They also discussed media recommendations for talking about suicide in the media.

**Conclusions:**

Sharing a personal story of hope and recovery may have a beneficial impact on the storytellers. Storytelling requires a careful preparation and training before going public and support and guidance is crucial in all stages of the storytelling, particularly to help unexperienced storytellers in going public and using their personal narratives to help prevent suicide.

**Supplementary Information:**

The online version contains supplementary material available at 10.1186/s12889-024-17661-4.

## Background

Media have a very important role in suicide prevention and working with media to increase awareness of suicide prevention has been one of the core components of many national suicide prevention programs and strategies [1, 2]. Specifically, guidelines for the reporting of suicide have been implemented in many countries to prevent sensationalized, suicide method-focused reporting which has been shown to be associated with subsequent increases in suicides, the so called “Werther” effect [3].

By far not all media stories on suicide are associated with harmful effects. In particular, media narratives of hope and recovery from a suicidal crisis have been shown to reduce suicidal ideation, termed the “Papageno” effect [4]. A recent meta-analysis has shown that individuals who are vulnerable to suicidal ideation or suicidal behavior especially benefit from this media material [5]. Some recent research further suggests that such portrayals might result in more help-seeking and reduce suicides [6].

The personal narratives of individuals with a lived experience of suicidal thoughts and suicide who publicly share their personal story of hope and recovery have been at the core of research on positive media potentials for suicide prevention. Such narratives have been found to be more clearly related to beneficial outcomes such as reductions in suicidal thoughts than other prevention messages [5]. Stories of individuals with a personal experience of suicidal ideation, suicidal behavior or the loss of a loved one appear to resonate better with vulnerable audiences, potentially because of their power to help establish a connection to audiences facing similar difficulties [7, 8]. These stories are also important, as they have the potential for a widespread influence, e.g., due to people sharing their stories on social media [9, 10].

Stories of hope and recovery might not only empower their audiences but might also affect the storytellers themselves. To the best of our knowledge, only one study so far has examined the perceptions of people with a lived experience who shared their story in public. This study involved twenty interviews with individuals with lived experience of a suicide attempt and/or bereavement due to suicide in Australia. The interviews revealed a number of beneficial effects for the storytellers. While the storytellers wanted to share their story in order to prevent others having to go through what they went through, they also noted therapeutic benefits for themselves. This included a feeling of creating a greater community and reducing feelings of stigmatization. However, the findings of the study also highlighted gaps in support for people with lived experience. Specifically, the participants noted some lack of support after going public with their story and feeling unprepared for coping with their personal vulnerability when confronted with triggering interactions with and reactions from their audiences [11].

The study was limited to Australia and therefore requires replication in other cultural settings [11]. On a more general level, it is known that sharing a personal story publicly renders a feeling of empathy with other affected people and presents a prosocial behavior by aiming to support and help others [12]. On the one hand, addressing personal suicidal thoughts and feelings might facilitate personal growth due to intensive engagement with (own) suicidality and its consequences on current living [8]. On the other hand, also negative effects of storytelling might exist and warrant scrutiny.

The previous study focused on the views of being a person with a lived experience as well as sharing their personal story of hope and recovery. Perceptions were assessed about the experience of going public and the expectations on how their story might impact others [11]. However, more information is needed on the whole process of storytelling starting from the idea of going public up to having had some experience of storytelling as well as what feedback people receive throughout the process and how this feedback impacted them.

The aim of the present study, therefore, was to qualitatively assess how storytellers in the USA have perceived the process of sharing their own story through media and public talks including the time after the publishing of their story (e.g., dealing with the feedback and the audience’s response to the story) in focus groups. We also used the groups to explore the different ways of sharing a personal story that were used and the perceived importance of media trainings directed at people who want to go public with their story. Specific aspects considered important regarding the process of sharing the story in media and public talks resulted in recommendations for future storytellers.

## Methods

We conducted three focus groups to assess the experiences and perceptions of suicide survivors of sharing their stories of hope and recovery in media and public talks. Focus groups are often used to facilitate an open discussion about sensitive topics and to explore differences between perceptions and experiences [13].

### Participants

Three focus groups with a total of *n* = 12 individuals of whom *n* = 5 had had suicidal ideation or a suicide attempt, *n* = 4 individuals who lost a close one to suicide, and *n* = 3 who experienced both were conducted. All participants shared their personal story via media and public talks. Media settings where stories were shared included articles and book chapters, podcasts and interviews, and live media such as television, radio or social media livestreams. We invited participants from the American organisation “Suicide Survivors United” (SSU) to take part in the focus groups. The board president of SSU, Dr. Sally Spencer-Thomas supported in the recruitment by distributing information about the study among the network and individually contacting people with who she was well connected or who would be particularly interested in this study. SSU is a renowned organisation for supporting individuals who want to share their personal story of hope and recovery on media. The organisation also provides media training to interested individuals. This training covers aspects on self-reflection and reflection on how the personal story could affect others. In this training, participants learn how to safely prepare and present a story of hope and recovery. This includes aspects on how to safely talk about suicide in order to prevent contagion effects using tools such as the media guidelines [14, 15]. Furthermore, trainees discuss how to build up a safety net of social contacts and are embedded in a social network where they receive support if needed.

At the time the focus groups were conducted, all participants had already had some experience with storytelling and had already developed their ways and strategies to cope with their personal experience of suicide, suicidal behavior and suicidal thoughts and feelings. Also, all participants were embedded in a supportive social network.

Table 1 shows a description of the composition of each focus group as well as the experience of the participants with different media settings based on self-report. Participants were assigned a number and a letter in order to keep anonymity. In the following, the participants’ quotes are referenced by their respective pseudonym (e.g., participant 3 from focus group A is being referred to as participant A3).

**Table 1:** Descriptive characteristics of the participants based on self-report

Focus group	Participant	Gender	Media setting of storytelling
**A**	1	Male	Public talks
	2	Male	Public talks, articles, livestreams
	3	Male	Public talks, articles, livestreams
**B**	1	Male	Public talks, videos, articles
	2	Male	Public talks, book (chapters), interviews
	3	Female	Public talks, TV, live media, podcasts, book (chapters)
	4	Female	Public talks, live media, interviews
	5	Female	Public talks
**C**	1	Female	Public talks, interviews
	2	Female	Podcast, recorded sessions
	3	Male	Public talks, articles
	4	Male	Public talks, interviews, recorded sessions

*Note*: Information derived from participants based on self-report

### Procedure

The focus groups were conducted in November 2022. All interested individuals were informed about the aim of this study and what their participation entailed beforehand. Of all the people who indicated their interest in participating in the focus groups, one person was unavailable at the stage of reaching out. Different days and times were suggested to give all individuals the chance to participate and people were grouped into focus groups based on their availability. Participants had the possibility to ask questions at any time. Written informed consent was sought before the focus groups were conducted. Each focus group lasted around 60 min and was conducted online using the meeting tool Webex Meetings. Group sizes were organised to hold four participants per group. Due to an illness, one participant could not participate in the organised group and attended one of the other groups. The main topics that were covered during the focus groups were:

- Motivation and reasons to do storytelling.
- Personal impact of storytelling and perceived impact of story on others.
- Media settings and media guidelines for safe portrayals of suicide.
- Recommendations for storytelling.

Groups were encouraged to talk openly about their process of sharing their story of hope and recovery on media and public talks. A semi-structured interview guide was used to guide the focus groups [see Additional file 1]. Probing questions were used in case the discussion stopped. Each focus group was recorded using the record function in Webex meetings.

#### Role of facilitator

All focus groups were facilitated by the first author, who is a female postdoctoral researcher at the Medical University of Vienna with a research focus on suicide and media. SK is experienced in facilitating focus groups with people with a lived experience of suicidal ideation and behaviour as well as suicide loss. Prior to the start of the focus groups, the facilitator had contact with each participant to clarify questions and concerns, and to express what they needed in terms of support. Participants were also encouraged to reach out and contact the facilitator after the focus groups if they needed support.

During the focus groups, the facilitator minimally contributed to the discussions and ensured that all topics of the interview guide were covered. Generally, the questions asked were broad and open in order to give the participants the possibility to fully express their views or recall personal experiences without being interrupted.

### Data analysis

All recorded focus groups were fully transcribed and read by both authors. We used thematic analysis to analyse the transcripts [16]. The transcripts were coded based on the research questions and subcodes related to additional statements. In a deductive approach, we used the interview guide as basis to code the topics that were addressed, which also represented our code families (e.g., motivation to do storytelling, personal impact and perceived impact on others, recommendations for storytelling). We also created codes based on topics that were repeatedly brought up by the participants, but which were not an explicit part of the interview guide (e.g., the need to hone down the story before going public, stigma, live media), resembling an inductive approach. The first author (SK) conducted the primary coding of the transcripts. The senior author (TN) screened those codings and coded a subsample of the focus groups independently. The coding was discussed between the authors afterwards, discrepancies resolved, and codes adapted.

**Fig. 1:**
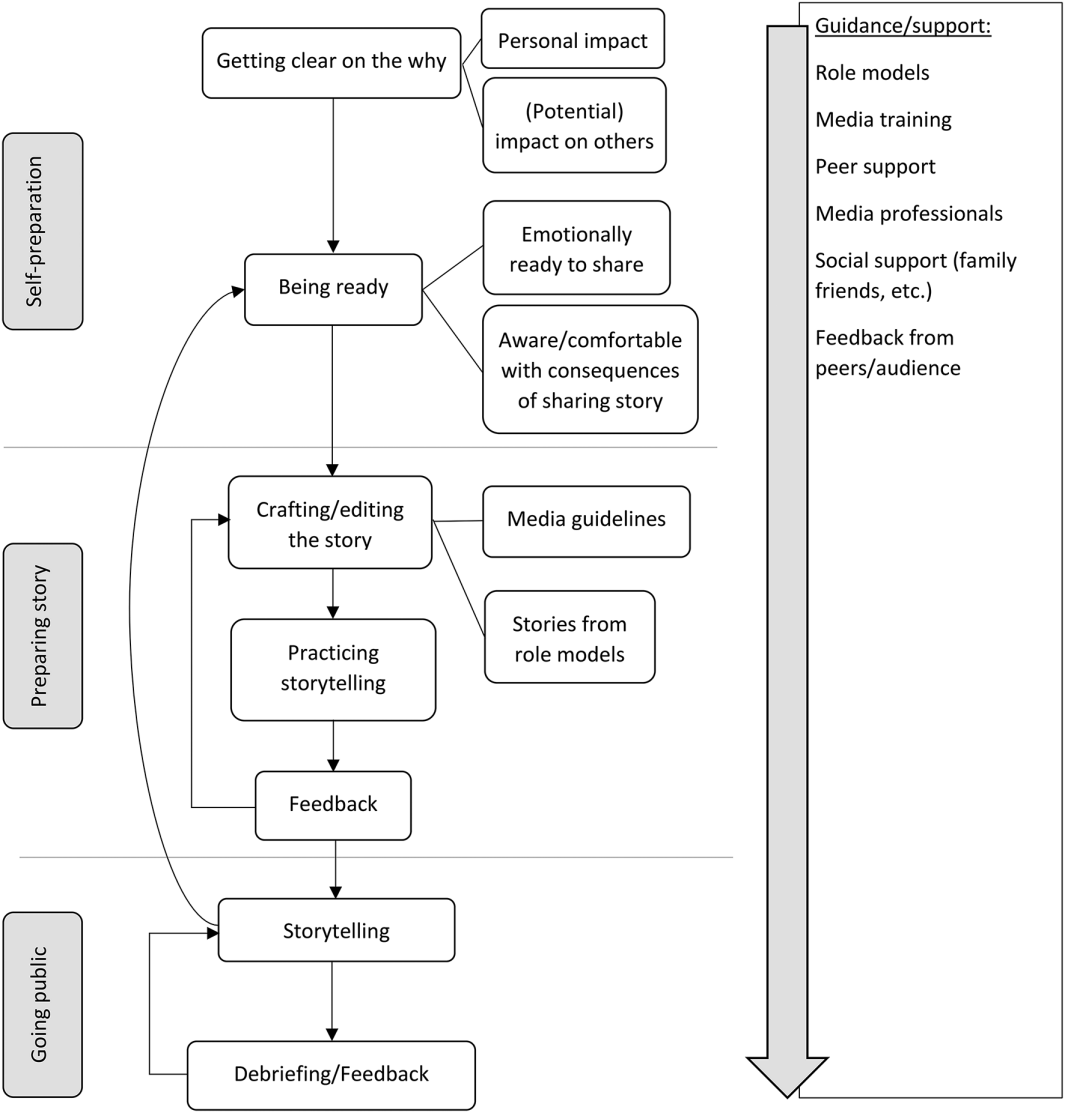
Process of sharing personal story via media and public talks. Factors influential to the process of sharing a personal story of hope and recovery in media and public talks or aspects recommended by the storytellers were collected to help new and unexperienced storytellers. A chronological order was created based on the participants’ noted chronology regarding the timing of these aspects

**Fig. 2:**
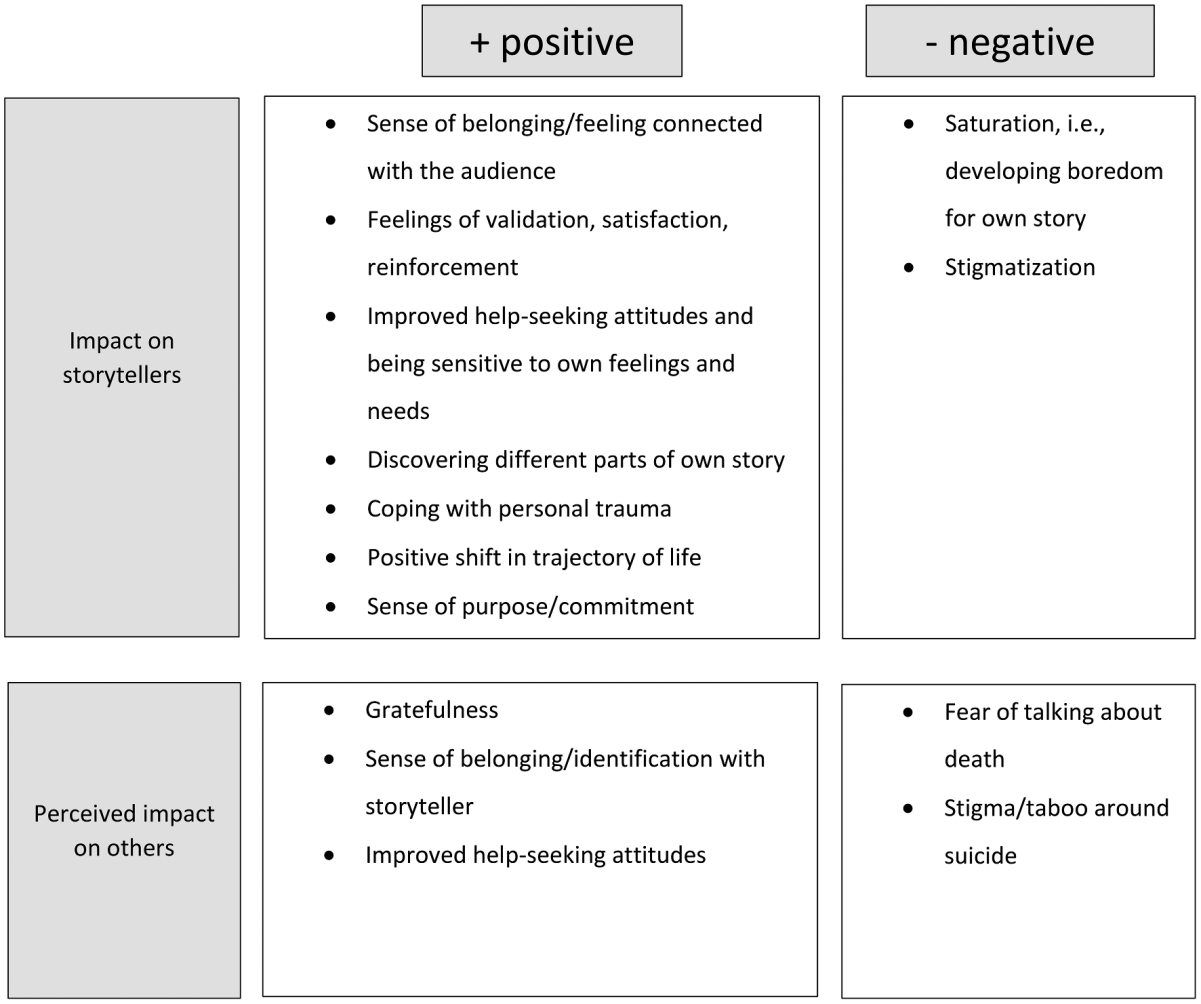
Impact of sharing story in public on storytellers and on audience. The impact is displayed according to whether it was framed positive or negative by the storytellers. Furthermore, the perceived impact is displayed separately for storytellers as well as for the audience

In addition, codes related to the perceived impact and recommendations for going public with their storytelling were summarised in two figures. In regard to the recommendations for the process of storytelling (Fig. 1), the codes were ordered in a chronological order as indicated by the participants. Codes related to the perceived impact of the storytelling were divided into positive and negative aspects, a connotation that was also based on the storytellers’ perceptions (Fig. 2).

Data saturation was assessed for the emergence of new topics brought up while conducting the focus groups, examining at which point themes were repeatedly mentioned in subsequent focus groups [17]. We found data saturation to be achieved after the third focus group for major themes, e.g., recommendations on doing the storytelling. New codes emerged particularly for specific topics such as the discussion of media guidelines or debriefing after the storytelling.

### Ethics statement

We obtained ethical approval from the Internal Review Board of the Medical University of Vienna (1379/2022). Written informed consent was sought for all participants prior to the start of the focus groups.

## Results

### Group and group dynamics

The group dynamics in all focus groups were harmonious. The storytellers appreciated hearing the other participants’ stories and expressed gratefulness for hearing their unique perceptions and opinions. The participants engaged in discussions with each other about their personal experiences. Agreement was expressed both non-verbally (e.g. by nodding or placing one hand on their heart) and verbally. There was no dissent between the participants in the focus groups. Participants who did not experience similar events as the ones that were described during a discussion said so and added their own views and experiences.

### Motivation and reasons to do storytelling

All participants agreed that being reflective about the reason or motivation to share their personal story was important in their process of storytelling (Fig. 1). There were different initial reasons for sharing personal stories of hope and recovery. This included both, intrinsic and extrinsic factors. While some participants were directly encouraged by other people to share their stories, others had role models who inspired them to do the same.

*[…] I think it came over time. Just watching other people and seeing, erm (…), the power and the freedom that brought to them and realizing that it was a, erm, it was a source of knowledge and knowing (…) and what it brought to people and that people were […] acknowledged for their experience […]*– participant B2.

Some participants initiated the process in sharing their story in order to cope with their personal trauma without any specific inspiration from other sources.

*I was a broken person who was going to die, erm, and to have an opportunity to (…), you know, really express the different narrative: one of struggle and one of effort but also one of, one of hope.*– participant C3.

At the core of motivation to do storytelling was the aim of helping others. Storytellers specifically expressed their wishes to provide hope and foster a sense of connection (Fig. 1).

*[…] but the whole desire was, because I want (…) other people to know that you can change your relationship to your own suicidal thoughts and that you don’t have to get to that place where you act on them.*– participant C2.

### Personal impact of storytelling

Regarding the personal impact of sharing the story, the participants reported a sense of belonging and feelings of validation, satisfaction, and reinforcement. Sharing their personal story resulted in changes in views in some storytellers regarding help-seeking and being sensitive to their own feelings and needs (Fig. 2).

*Now I think of like man, […] I gotta take care of myself, […] I gotta make sure I talk to people, I gotta make sure I go and get help. […] I would say before I was a little worried about what people were gonna do to judge me if I ever (…) act like this or I ever looked to seek help […] I value my life and everybody else and life is more valuable to talk about it than not to talk about […]*– participant C4.

The long-term effects of storytelling on themselves were perceived differently by the storytellers. While some participants reported that they discovered different parts of their story in the process or noticed an improvement in coping with their trauma, one participant reported to have developed a feeling of boredom (or saturation) regarding his personal story (Fig. 2).

Sharing the personal story was also associated with some negative experiences in some participants. Particularly, some storytellers experienced people from the audience being very worried about them and asking them if they were okay which was sometimes perceived as unpleasant and stigmatizing as it made them feel vulnerable and fragile rather than empowered and supported (Fig. 2).

*[…] when you say the word suicide, right, they’re immediately going to, like, how can I help this person, and, and they’re trying to maybe (…) not judge you but gauge, you know, is he, is he healthy, is he (…), you know, how’s, how’s his mood, you know, stuff like that.*– participant B1.

One participant recalled her experience of being treated differently by some colleagues after having disclosed her personal experience of suicidal ideation. These colleagues were especially concerned after she wanted to invite other suicide survivors to their organization.

*What I felt like I experienced stigma and peop-, some people changed the way they treated me, and erm (…), few were concerned about ha-, me having the team [a team of other suicide survivors] meet at the organization […] Somebody said like what if they bring a gun […] it did not feel very good […]*– participant B4.

Some participants felt a sense of purpose or commitment through the experience of sharing their story. Some even reported that storytelling changed their live trajectories for the better (Fig. 2).

*[…] I had no idea when I started telling my story how that would shift the trajectory of my life, erm, both professionally and personally and I think that that is one of the unexpected joys of a hard thing […] you know, the act of sharing that story […] and the response to that but also, again, encouraged me to do more, erm, and that has opened up so many wonderfully and joyous things in my life […]*– participant C3.

### Perceived impact of story on others

The participants talked about the perceived impact of their personal story of hope and recovery based on feedback and reactions from the audience they received, mostly without differentiating between the specific media settings. Regarding immediate feedback the storytellers received from the audience, participants reported that individuals providing feedback on their storytelling felt grateful for the stories and described a sense of belonging and a feeling of identifying and empathizing with the storyteller (Fig. 2). One participant specifically recalls feedback she received after a podcast interview:

*[…] but the feedback that I was getting from them was like […] that what I shared really spoke deeply to them and that (…) it helped them see that it could be different for them, too, and that they wanted to know more, they wanted to learn more […]*– participant C2.

Some participants also talked about experiencing feelings of fear in the audience and stigmatization of speaking openly about topics such as death or suicide in some parts of the audience (Fig. 2).

*[From my storytelling] I often (…) get a better picture of certain communities and how they may deal with or not deal with it or how Western culture does not do well with death at all, especially suicide. Or very afraid of it.*– participant A1.

The storytellers described that they also got feedback from their audience indicating an improved willingness to seek help and raised hope that this might result in their audience offering help to other people in need (Fig. 2).

*Now he’s like I look at everything different. So he said, […] he understands now […] so hopefully he’s learned from it, now he’s gonna teach his guys […] maybe help save somebody’s life sometime, you never know, so (…)*– participant C4.

### Media settings and media guidelines for safe portrayals of suicide

Participants agreed that no specific media setting was superior to another in terms of relevance for storytelling, but they noted that different ways of sharing their personal story allowed for different experiences and audiences.

The participants described that public talks offered a stronger sense of intimacy, although it was only possible to serve a limited audience at a specific scheduled date/time. On the other hand, print articles allowed for repeated reading. The preferred way for storytelling was dependent on the storyteller’s personal preference (e.g., written word vs. public speaking) and was also contingent upon specific opportunities that arose to share the story (e.g., being invited for an interview).

Several caveats and negative experiences were shared specifically related to live media and social media. Live interviews were described as somewhat risky by potentially losing control of your own storytelling or by having your words twisted around.

*I had that, I had on live television, I had someone say and now we’ll take some callers from people who are suicidal, I’m like, oh no, we’re not, we’re, we’re not doing that on live television and they did and it was like, you know, a pre-, pretty provocative news station, I should have known better. Anyway (…), but crazy things can happen on live television.*– participant B3.

In light of having no control over their storytelling, one participant also shared a negative experience related to social media.

*[…] I think social media can be hard, er, because once you put it out there then all of the feedback is live (…) for everybody to see (…) and you have no control over that and we just saw, you know, one of our beloved colleagues get railed on twitter this week and it’s just heartbreaking. Erm, and there was nothing she could do, you know, erm (…), so (…), I think that’s where social media can be ki-, risky.*– participant B3.

In the context of media training, media recommendations for talking about suicide in media were brought up by the participants and discussed in the group (Fig. 1). Specifically, some participants were confused as to what was regarded safe or not in storytelling. In particular, regarding the pros and cons of mentioning the method of a suicide attempt in the personal story, one participant originally wanted to mention the suicide method, but during media training was advised against doing it and eventually refrained from it. Another participant, contrariwise, was confronted with a news media journalist who urged him to include the method in the newspaper article, which he did not want in the beginning but eventually agreed and was happy about it in the end.

*[…] specifically I’ll just call that one thing which was something that I never thought I would do when I started which was to actually describe how I was gonna kill myself, erm, but the, the editor at the paper (…) in the last round of edits was very insistent that I needed to disclose the fact, erm, because she felt it would create and generate more connection and authenticity and I think also potentially for the readers, erm, you know, feel a sense of urgency and kind of realness about like how close one can come to, to end one’s life, but I would not have done that if I had been on my own, so I think that there is something very powerful about engaging with an editor or someone else on the page […]*– participant C3.

*[…] I wanted to share I thought about killing myself in this particular way, in this particular and name very specific method, because it’s very important to my own healing journey and they all meant something different for me, erm, but then had to rework it not to do that and then to hear that, well, actually, I was encouraged to add more detail, it’s like, well this is interesting. […] I wonder if maybe there’s too much fear around talking specifically about methods.*– participant C2.

In this regard, the two participants discussed the use of media guidelines and did not necessarily agree with the recommendation to avoid the portrayal of suicide methods as they believed it would resonate more with the audience. For the majority of participants, however, this did not appear to be an issue and they did not highlight any relevance of adding the suicide method. Overall, there was a great consensus in the group that storytellers wanted to provide safe messaging.

*“[…] knowing that there was a workshop in our area where people talk to suicide loss survivors that have told their story safely. Safely for them and safely for others. Erm, it’s knowing some of these guidelines, like, and very helpful to sort of learn, er (…) from Sally [board president of SSU, who also leads media trainings] this […]–* participant B2”.

### Recommendations for storytelling

The participants were asked if they had any recommendations for others who would like to share their personal stories of hope and recovery. All aspects that emerged are chronologically ordered below according to aspects prior to and during as well as in the period following the storytelling (Fig. 1).

Participants agreed that emotional readiness was key. Specifically, some participants highlighted the importance of outweighing the consequences of sharing a personal story to avoid triggering suicidal thoughts and feelings in themselves.

*[…] I would recommend like (…) go-, going to the depths of all of it and like uncovering and tracing like what it, what it means to you in this present day and then, like, going after, going through that process with yourself, identifying like what needs to be shared now. […] doing that process was really helpful for me to feel like there weren’t any like sharp surprising edges to my past that were going to like trigger or activate me like anymore, erm (…) and that I could feel and trust myself to go and share about some of these, erm (…) scary and dark parts of my life without, erm, without worrying about me in the present day.*– participant A3.

Emotional readiness was not only seen as important prior to the storytelling but was rather brought up as an ongoing process (Fig. 1). One participant reported that, even after having shared his story several times, it was still painful for him to tell his story and to relive his experience all over again. In order to cope with this, he adjusted his approach by reading his story off paper instead of speaking freely.

*[…] what I have learned to probably do is, erm, (…) when I go to give my talk I, I just, I read it off paper […] I don’t try to go back and rehearse it […] because I can’t. It’s, it’s, the story is too painful, and (…), be-, the first few times that I went to talk about it, I tried to rehearse it and, you know, I, sometimes I’d rehearse it 25 times or 30 times. Every time it would get to a point where it would just be (…) miserable […]*– participant A2.

At all stages of the storytelling, support and guidance were described as crucial, and participants emphasized the importance of not going through that process alone. Peers (e.g., other suicide survivors) as well as role models who already shared their story were frequently mentioned as a source of guidance as well as family and friends but also media professionals such as editors or interviewers (Fig. 1).

*[…] I had no clue how to start, how to get it going and seeing their stories develop and watching how they did it and they created like, I was just amazed by how wonderful their, their stories were and how, ho-, it helped me get my story out […] I don’t think I would have been anywhere near (…) to where I was when I got my story done and out if I didn’t have them, you know, leading the way, and kind of giving me their examples […]*– participant C4.

Having support also included practicing the story in front of others before going public. In this regard, participants frequently mentioned friends or trustworthy people to practice in front of (Fig. 1).

*“I would suggest, erm (…), practicing your talking about your own story in many different ways, in many different contexts with people, with safe people, to really get comfortable sharing it so that you can know inside that when you share something that you’re not going to end up getting overwhelmed by that thing that you’re sharing […]*– participant C2”.

Crafting and honing the story and knowing which parts of the story should be shared was considered beneficial and considered a dynamic process. Specifically, some participants noted that their story changed through time due to their own perception and needs but also due to feedback from the audience. Participants also agreed upon the importance and helpfulness of media trainings in developing and honing down the personal story (Fig. 1). This was considered particularly relevant regarding live media.

*Absolutely essential, if you’re gonna be on any live situation. Absolutely essential. Because there are definitely things you can do to tell the story you wanna tell. You don’t have to answer the questions, you can easily learn how to pivot (…), move the story over (…) say, you’re not gonna answer it [laughs] you know, there’s lots of things you have power as somebody being interviewed that you don’t realize you have if you don’t go through that training and like practice a little bit […]*– participant B3.

Concerning the decision on how to share the story and which media setting to choose, having control over your own personal story and/or a trustworthy relationship with the media professional was frequently mentioned as crucial aspect.

After going public and doing the storytelling, participants emphasized self-care and de-briefing as some participants reported feelings of being overwhelmed by exposing their vulnerability in front of others. This sentiment, however, was not shared by everyone (Fig. 1).

*[…] you know, I, I, I know how to do it, and I can, I, I know how far to go and all of that stuff, but it is also exhausting, erm, so I would say, another suggestion would be to have an aftercare plan, er, because a lot of times, you know, again if you’re speaking to a large audience, you’re gonna have a lot of people coming up, they’re gonna ask questions, you know, you’re gonna be on for a little bit while, and then you just go lie down, something, like you should go, create a quieter, er, place to debrief. I, I, (…), I, I, I get very tired after especially large presentations, ‘cause it takes a lot of energy, erm, but I think sometimes, er, you know, it’s hard, it’s hard to tell the difficult story over and over and over and over and over and over and over.*– participant B3.

## Discussion

Storytellers perceived the sharing of their own story as a mainly positive experience for themselves and also for their audience based on the audience feedback they had received. The decision to do storytelling via media and public talks was found to benefit from a process of careful personal preparation (to be emotionally ready) and practice. Support from various sources was perceived to be crucial in all stages of this process. This included media training to help new or unexperienced storytellers in going public with their stories, especially when doing storytelling in live media.

### Contextualization with other research

Engaging with people with lived experience in efforts to prevent suicide is not new and has been defined as a way forward for suicide prevention [7]. One of the motivations for storytelling is the aim for connection and building a community. While sharing a personal story of hope and recovery was reported to lead to a feeling of belonging among the audience [11], storytellers in our focus groups reported that it also had the same effect on themselves which was highly valued as it rendered a sense of being part of a broad community and not being alone due to the realisation that other people were going through similar situations.

However, sharing a personal story of hope and recovery was also associated with some specific negative experiences. Particularly, stigma is an important aspect in storytelling and suicide remains to be a stigmatized topic which affects families, friends and professionals working in the area. Suicide stigma puts people with experience of suicidal ideation, suicide attempt or bereavement at elevated stress, as they often experience negative reactions from their environment, but also a personal stigma towards themselves or not feeling understood [11, 18, 19]. Efforts to reduce stigma appear to be part of the motivations for doing storytelling for some storytellers [11]. Although storytellers did not explicitly express this in the present study, they frequently reported being confronted with taboos of speaking about death and dying. Furthermore, the focus group discussions also revealed that sharing a personal story of hope and recovery often leads to (uncomfortable) out-of-place questions about the storyteller’s well-being from the audience, making some storytellers feel stigmatized. The sometimes negative and distressing experiences of storytellers speaking about deeply personal issues in a stigmatized area highlight that an open conversation still proves to be sometimes difficult. In traditional media, stories of suicide death still clearly outweigh stories of hope and recovery, which might have the best potential to support other individuals in crisis and considering suicide [4, 5].

Sharing a personal story of hope and recovery was reported by storytellers to be an ongoing process. Specifically, the focus and scope of the story was perceived to shift over time, and thus the story regularly adapted [11]. Storytellers mentioned that this was partly due to regularly receiving feedback from the audience following their sharing of the story, which encouraged them to open up more, but also due to their personal growth and recovery journey.

The process of sharing a personal story of hope and recovery sometimes appeared to reveal vulnerabilities of storytellers that they had not been aware of before their storytelling. Some storytellers reported feeling emotionally triggered by reactions and experiences shared by their audience with them after their storytelling, which they did not anticipate and which put them in some distress [11]. In order to help cope with such experiences and prepare for them, social networks are needed to help storytellers at all stages, i.e., before and after going public. The focus group discussions clearly indicated that storytellers who were generally integrated in a safety net felt better prepared.

In this regard, the different media settings and their potential advantages and disadvantages need to be closely considered. Storytellers highlighted specifically the potential dangers of live media or social media, e.g., by losing control of one’s own story. Due to its easy accessibility, social media gives people the possibility to share their story quickly as compared to other media settings [9]. Risks include, that messaging can quickly spin out of control [20]. Storytellers, especially those without relations to other storytellers, any access to trainings or specific experience with this setting need to be aware of these consequences and have to be adequately prepared to cope with such situations. Especially, media training was deemed essential by the participants, and online trainings or storytelling workshops are available to support new and unexperienced storytellers.

With regard to safe messaging for suicide prevention, recommendations about the messaging of suicide in news and fictional media have been established to ensure safe content for the audience and prevent imitation effects [14, 15]. While most focus group participants were aware of the guidelines and generally appeared to follow them, there was also some ambivalence and confusion towards these guidelines. Specifically, one storyteller wanted to mention her specific suicide method but was advised not do it, whereas another participant was encouraged by a journal editor to do so. The reasons for this ambivalence appeared mixed, with some reasons being related to contemplations about a potentially beneficial impact on the audience by creating more connection, and other reasons related to the storyteller’s personal desire of coping with trauma. These considerations suggest a field of tension between, on the one hand, a desire to mention the suicide method among some storytellers, which, from their perspective, was a relevant part of authenticity, and, on the other hand, a desire to do safe messaging that does not harm others. Besides informing the participants about the existence and content of the media guidelines, media trainings should also highlight the reasons for these guidelines to make sure storytellers are aware of the reasons why the portrayal of suicide methods should be avoided.

There is good evidence about the harmful impact of media reporting of suicide particularly with regard to the reporting of celebrity suicide [1, 3]. Also, social media, such as suicide message boards can have harmful effects [21]. There is no clear evidence, however, on how the positive effects of stories of hope and recovery might be affected by the inclusion of a specific method (as opposed to stories about suicidal behaviors). It remains to be tested if specific story characteristics, such as mentioning the method, could possibly have a different effect depending on the overarching storyline, i.e., a suicide death story or a story of hope and recovery.

### Strengths and limitations

A strength of this study includes the wide range in the study participants’ storytelling experience ranging from live broadcast to public talks, thus covering a broad spectrum of perspectives and opinions. Data saturation was achieved for major themes.

A limitation of the study represents the lack of generalisability and representativeness of our study sample. We only conducted the study with people based in the United States of America, and we only included one setting, Suicide Survivors Internationals, which means that only selected individuals with at least some network around them and some preparation and training were included in the discussion. Therefore, we cannot generalize findings to the experiences in other settings or experiences of storytellers without any media training or who might not be embedded in a safety net (e.g., people who would like to share their story on social media).

Furthermore, the reported effects on the audience are based on anecdotal evidence only. Storytellers shared their perceptions on how they think their story impacted the audience based on the specific feedback they received, but there was no systematic assessment of audience feedback among members of the audience. Regarding the long-term effects of sharing a personal story of hope and recovery, not much is known yet. The long-term effects might be different from the short-term effects as reported by the participants in our focus groups and thus need to be investigated in more detail in future studies.

## Conclusions

Sharing a personal story of hope and recovery appears to have many positive effects on the storytellers themselves. The findings, however, also show that going public with one’s own story of hope and recovery requires a long process of careful preparation and practice beforehand. Social support, and reflection and adaptation of the story throughout the process of storytelling were deemed crucial.

### Electronic supplementary material

Below is the link to the electronic supplementary material.

**Supplementary Material 1:** This file entails the interview guide that was developed for this study and used to collect the data

## Data Availability

The questionnaire that was developed for this study can be found in the additional material 1. The data that support the findings are available on reasonable request from the corresponding author [S.K.]. In order to protect the privacy of the participants, the transcripts are not publicly available.

## Abbreviations

SSU: Suicide Survivors United
