# Supplementary material for: Experiences of suicide survivors of sharing their stories about suicidality and overcoming a crisis in media and public talks: a qualitative study
Source: BMC Public Health. 2024 Jan 10;24:142. doi: 10.1186/s12889-024-17661-4 (PMC10777649; doi:10.1186/s12889-024-17661-4)
Supplement: Supplementary file 1 — Supplementary Material 1: This file entails the interview guide that was developed for this study and used to collect the data [file 12889_2024_17661_MOESM1_ESM.docx]

Study materials

This file entails the interview guide that was developed for this study and used to collect the data. The interview guide contains briefing instructions for the facilitator as well as the questions used in the focus groups and probing questions in case the discussion came to a halt.

Interview guide for focus groups

Group: ______________________ Date:____________

**Briefing:**

- Declaration of consent
- Start audio recording
- Thank participants for their study participation
- Inform participants about aim and purpose of the focus groups
- Emphasize that the aim of conducting focus groups is not being in perfect agreement with the other participants, but rather sharing unique and different perspectives and opinions; every opinion and every perspective is welcome; there is no right or wrong
- Promote an open discussion, encourage free speech; only interrupt when necessary (e.g. a person does not get to share their part); interviewer stays in the background
- Inform about duration and structure of the focus groups
- Any open questions?
- Start with asking people to introduce themselves
  - Name, gender, age, what is the story about (suicide attempt survivor, past suicidal ideation, bereavement from suicide), conflict of interest
    - have you had any commercial interest in sharing your story?

**Your story:**

Let’s start with you telling me about the process of how you decided to share your personal story with the public.

- What is the main narrative and focus of your story?
- What was important in your decision to share your story?
- Which media types or media type have you used to share your story?

What was the feedback you have received following the sharing of your story and who gave you feedback?

- Feedback from suicidal individuals
- Feedback from others in difficult life situations
- Did you also receive negative feedback?
- Effect of feedback on yourself? Positive and negative effects?

Can you tell me more about your experiences with sharing your story in the specific type/the types of media you have used? Are there any differences or advantages or disadvantages regarding the use of the specific media type?

In hindsight, is there something that you would have done differently now with all the experience you have gained?

What did you need or would have needed in your preparation phase? Anything that you would do different in terms of preparing now with your experiences gained?

What would you recommend others who want to talk about their suicidality or overcoming a crisis in media that they should consider and do in preparing for sharing their story in media?

- any specific recommendations in terms of
- personal preparations / self-reflection,
- decision-making to share or not share a story ;
- decision-making on the selection of specific aspects of one’s story for sharing: where should the focus be put in a suicide preventive narrative;
- selection of specific target audience,
- selection of specific media types?
- how should the story be shared with the public?
- Any other recommendations?

What do you think about the relevance of…

- media training and
- self-reflection before telling the story?

**Other:**

Is there something else that you feel is important to say in this focus group but which we haven’t touched upon yet?
